# Positive residues of the SARS-CoV-2 fusion domain are key contributors to the initiation of membrane fusion

**DOI:** 10.1016/j.jbc.2024.107564

**Published:** 2024-07-11

**Authors:** Daniel Birtles, Lijon Guiyab, Wafa Abbas, Jinwoo Lee

**Affiliations:** Department of Chemistry and Biochemistry, University of Maryland, College Park, Maryland, USA

**Keywords:** SARS-CoV-2, membrane fusion, membrane lipid, electrostatics, ^19^F NMR, virus entry, Lipid-Protein interaction, mutagenesis

## Abstract

SARS-CoV-2 is one of the most infectious viruses ever recorded. Despite a plethora of research over the last several years, the viral life cycle is still not well understood, particularly membrane fusion. This process is initiated by the fusion domain (FD), a highly conserved stretch of amino acids consisting of a fusion peptide (FP) and fusion loop (FL), which in synergy perturbs the target cells' lipid membrane to lower the energetic cost necessary for fusion. In this study, through a mutagenesis-based approach, we have investigated the basic residues within the FD (K825, K835, R847, K854) utilizing an *in vitro* fusion assay and ^19^F NMR, validated by traditional ^13^C ^15^N techniques. Alanine and charge-conserving mutants revealed every basic residue plays a highly specific role within the mechanism of initiating fusion. Intriguingly, K825A led to increased fusogenecity which was found to be correlated to the number of amino acids within helix one, further implicating the role of this specific helix within the FD’s fusion mechanism. This work has found basic residues to be important within the FDs fusion mechanism and highlights K825A, a specific mutation made within the FD of the SARS-CoV-2 spike protein, as requiring further investigation due to its potential to contribute to a more virulent strain of SARS-CoV-2.

Although the threat of SARS-CoV-2 has diminished significantly since the height of the COVID-19 pandemic, there is still much to be learned regarding the molecular basis for the high pathogenicity. Enveloped viruses contain glycoproteins embedded within their membranes that are responsible for catalyzing infection and in the case of SARS-CoV-2, this is the spike (S) glycoprotein (1). The process of infection can be split into two distinct actions: receptor binding and membrane fusion, which are facilitated by the S1 and S2 subunits, respectively (2). A combination of receptor binding and cleavage at the S2′ site leads to dissociation of the S1 subunit and significant conformational changes within the S2 subunit. Such structural changes are responsible for propelling the fusion domain (FD), found at the N-terminus of the cleaved S2 subunit, into the lipid membrane of the target cell. At this point, the virus is anchored in both the viral and adjacent target cell membrane *via* its transmembrane domain and FD, respectively. Following insertion of the FD into the target membrane, heptad repeats 1 (HR1) and 2 (HR2) of the S2 subunit fold back upon themselves to bring the opposing membranes into close proximity before they eventually coalesce (3). This process, known as the six-helix bundle mechanism, is characteristic of class I viruses and provides the favorable free energy landscape necessary for successful fusion events (4, 5).

SARS-CoV-2 is understood to be able to enter host cells *via* membrane fusion at either the plasma membrane or the endosomal membrane, a unique trait that is thought to aid in the success of viral infection (6, 7). One key contributing factor to this ability is the presence of the proteolytic enzymes transmembrane serine protease 2 (TMPRSS2) in the plasma membrane and Cathepsin L in the endocytic pathway, both of which can cleave S2′ to create a fusion-ready glycoprotein on the viral surface. Furthermore, a preference for the SARS-CoV-2 FD to fuse under environmental conditions similar to that witnessed in the endocytic pathway has been established. Both *in vitro* and *in vivo* work exhibited an increase in fusogenecity at a pH below neutral (≤pH7) (8, 9), as well as a positive and specific relationship with the endosomal resident, anionic lipid bis(monoacylglycero)phosphate (BMP) (10). Specific pH environment, enzyme localization, and membrane lipid composition are all common triggers associated with the initiation of the fusion process for several different viruses (11). A greater understanding of the SARS-CoV-2 FD’s molecular mechanism regarding the initiation of fusion *via* its unique FD is necessary to fully understand how such triggers lead to a successful fusion event.

The coronavirus FD has previously been shown to contain two distinct regions: a fusion peptide (FP) and a fusion loop (FL), with both capable of fusing independently but significantly more effective in synergy (12, 13). This concept of two synergistic domains within a single fusion subunit is unique and suggests a novel molecular mechanism. Although initially thought to only exist within the coronavirus family, a very similar FD with seemingly two distinct regions was recently discovered in the Lassa virus, a prominent member of the Arenavirus family deemed to have pandemic potential by the World Health Organization (WHO) (14). Alongside the unique impact of the two fusogenic domains, the SARS-CoV-2 FD also contains several charged residues spread throughout, four positive and six negative. This is in stark contrast to other viral FDs such as those found in Influenza and HIV which contain two and zero charged residues respectively (15, 16). While the formation of an internal salt bridge has been ruled out (14), and several acidic residues have been implicated in binding Ca^2+^ (17), little research has focused on the significance of the positively charged residues. A structure-based approach using a combination of NMR and molecular dynamics found that K825 and K835, found in the two helices of the FP, were buried within a bicelle environment, snorkeling to place their charges amongst the lipid headgroups (18). Alternatively, while the positive charge of R847 was also found within the headgroup region, the position of the backbone and the aliphatic chain was dictated by the dynamic movements of the FL (18). As anionic lipids have been found to be crucial for the fusogenecity of the FD (10), it is reasonable to assume that complementary interactions between those lipids and basic amino acids within the FD could be crucial for the underlying molecular mechanism to initiate fusion.

Here, we aim to elucidate the importance of four well-conserved (19), positive amino acids found within the SARS-CoV-2 FD (Fig. 1). Within the 40-amino acid FD construct, both alanine and charge-conserving mutants for all positive amino acids (K825, K835, R847, and K854) were created and expressed, with the exception of K854A. Each mutant perturbed the function of the FD, with both R847 mutants displaying the greatest loss, whilst K825A increased fusogenecity. Moreover, an increase in α-helicity for K825A was found *via* CD, with all other mutants displaying the same global secondary structure as Wt. To understand the role of the basic amino acids further, we employed a ^19^F-Phe labeling approach to gather atomic resolution structural data. After assigning the ^19^F-Phe Wt peaks through a mutagenesis-based approach, chemical shift perturbations (CSPs) were plotted for each mutant against Wt. K825A displayed the only significant CSP, corresponding to a ^19^F-Phe labeled residue (F823) in helix one and this was confirmed as the site of the α-helical extension *via* traditional ^13^C and ^15^N backbone assignment for K825A. In summary, all four basic residues play a part in the fusion process, with K825 in helix one of the FP and R847 within the FL appearing to have the most important roles. Together, the data suggests that electrostatic interactions between positive amino acids within the FD and negatively charged lipids in the target membrane could be important for the molecular mechanism underlying the initiation of SARS-CoV-2 fusion.

**Figure 1:**
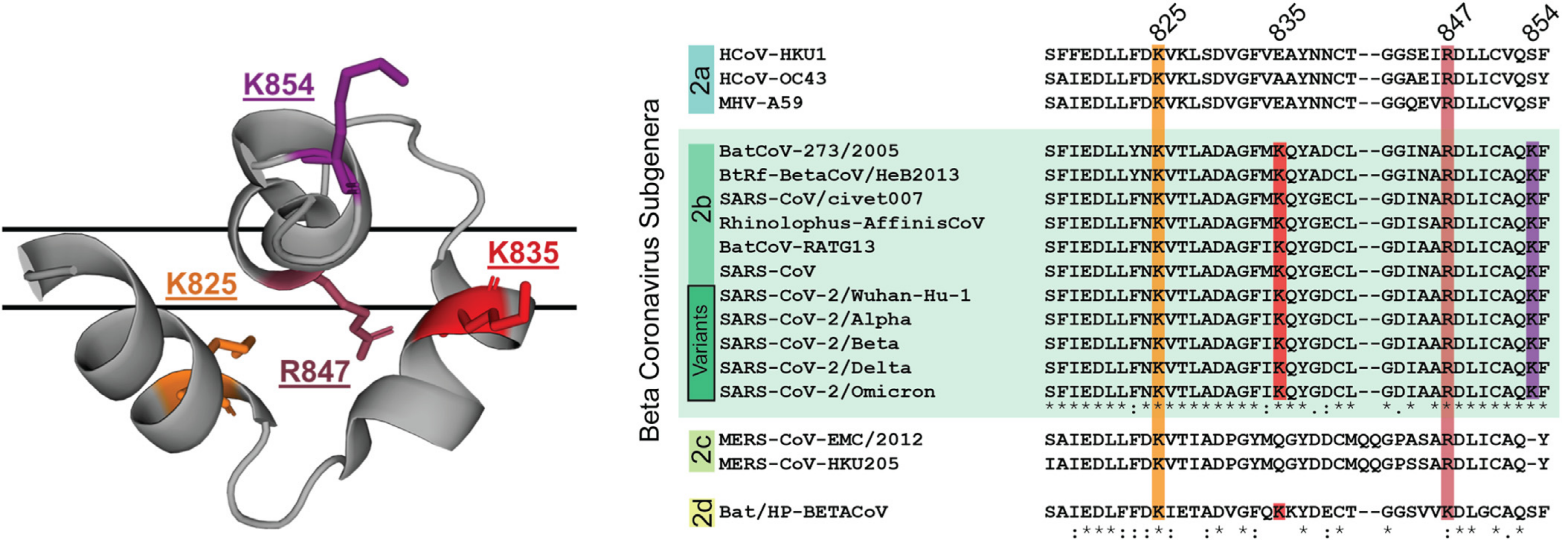
**The structure of the SARS-COV-2 FD embedded in a DMPC:DH**^**7**^**PC bicelle (PDB:****7MY8****), with the basic amino acids highlighted and sequence conservation across the four beta coronavirus subgenera.** Alignment was created using Kalign at EMBL-EBI^19^.

## Results

A preference for the SARS-CoV-2 FD to initiate fusion with membranes containing anionic lipids has implicated the importance of four positively charged amino acids within the FD’s molecular mechanism. A total of eight mutants were created, consisting of changing a positive residue to alanine and also maintaining the positive charge: K825A/R, K835A/R, R847A/K, and K854A/R. All mutants were expressed *via* an *E.coli* system except K854A which did not express at all. The locations of the basic residues’ side chains were identified within a zwitterionic bicelle and are displayed in Figure 1, along with the high level of conservation for each residue. Within this structure, K825, K835, and R847 sidechains can all be found inside the bicelle in proximity to the lipid headgroups, potentially implicating them in crucial interactions for the structure and/or function of the FD (18). On the other hand, K854 is found in solution, facing away from the bicelle.

**Figure 2:**
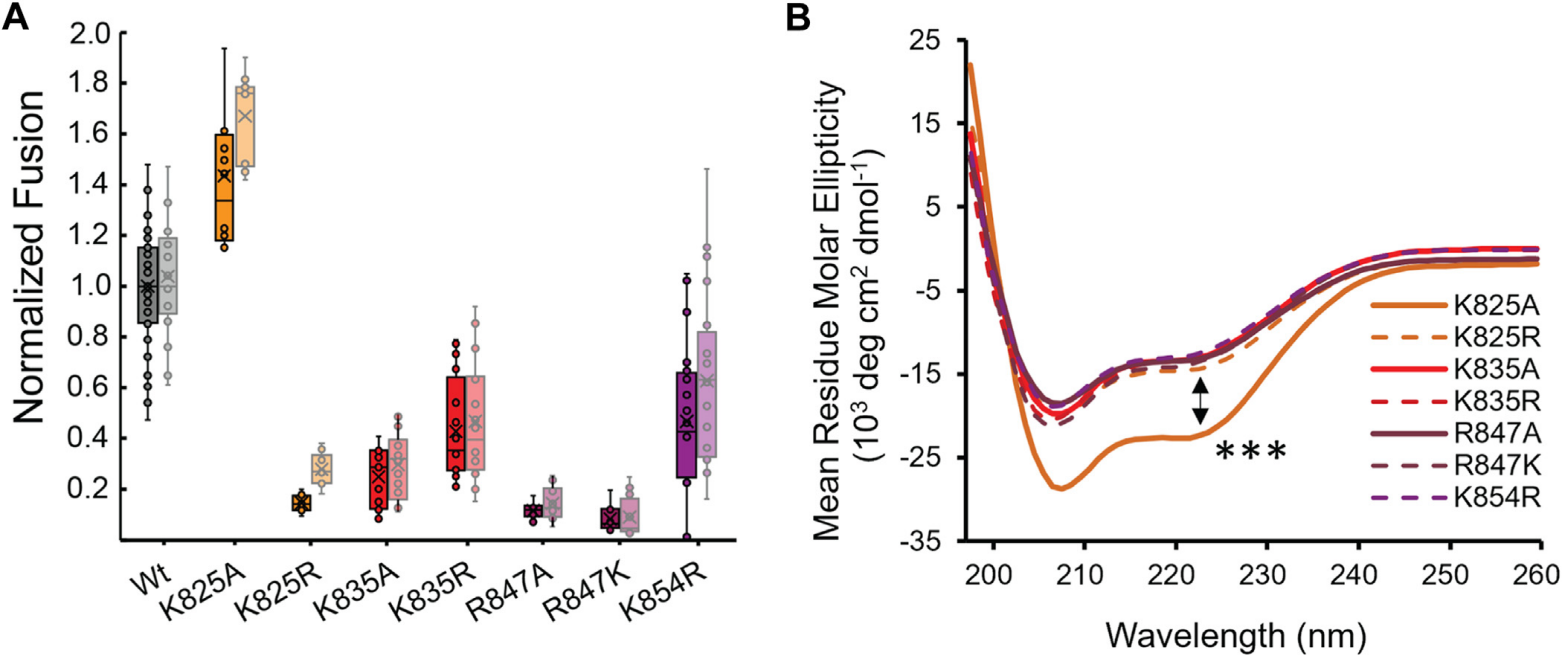
**Functional and global secondary structure analysis of the FD mutants.***A*, the same general relationship is witnessed for the fusogenecity of each mutant, regardless of the anionic lipid present (80:20 POPC:BMP = Full, 80:20 POPC:POPS = Opaque). K825A results in a gain of function whilst all other mutants result in a loss of function, with R847 particularly sensitive. Individual data points represent technical replicates as well from several independent experiments. *B*, through CD in DPC micelles, K825A displays a significant increase in α-helicity whilst all other mutants show a similar global secondary structure to Wt which has been published previously.^12^ A one-sample *t* test was employed with K825A used as the standard and the MRE value at 222 nm compared to the mean of all other mutants, (t_0.001, 5_ = 6.87 < t_calculated_ = 28.66).

To understand the role of individual positive charges on the fusogenic properties of the FD, we first sought to measure the impact of each mutant *via* an *in vitro* fusion assay with membranes containing POPC (50 mol%) and either BMP or POPS (50 mol%). The reason for independently assessing the two anionic lipids was to understand whether the previously discovered preference for BMP was due to any specific interactions with a particular basic residue. When standardizing the results to Wt with the respective lipid composition, the same trend is witnessed for both anionic lipids suggesting no preferential interactions take place (Fig. 2*A*). Within these trends, we see that K825A results in a gain of function/fusogenecity, whilst all other mutants resulted in a significant loss of function/fusogenecity, with R847 mutants displaying the least amount of fusion (Fig. 2*A*). Alongside fusion, the affinity of the initial interaction between the FD mutants and the anionic membrane (POPC:POPS, 50:50 mol%) was perturbed (Fig. S1). Interestingly, both K825 and R847 were the most significantly impacted by the alanine mutation. Moreover, when analyzing the structure of the mutants *via* CD in a DPC micelle environment, K825A displayed a significant increase in α-helicity compared to all other mutants which were similar to Wt (Fig. 2*B*). Hence, K825A displays an increase in α-helicity that appears to directly correlate with an increase in fusogenecity, while a lack of change in the CD spectra from Wt suggests changes in secondary structure are not responsible for the loss of fusogenecity witnessed across all other mutants.

**Figure 3:**
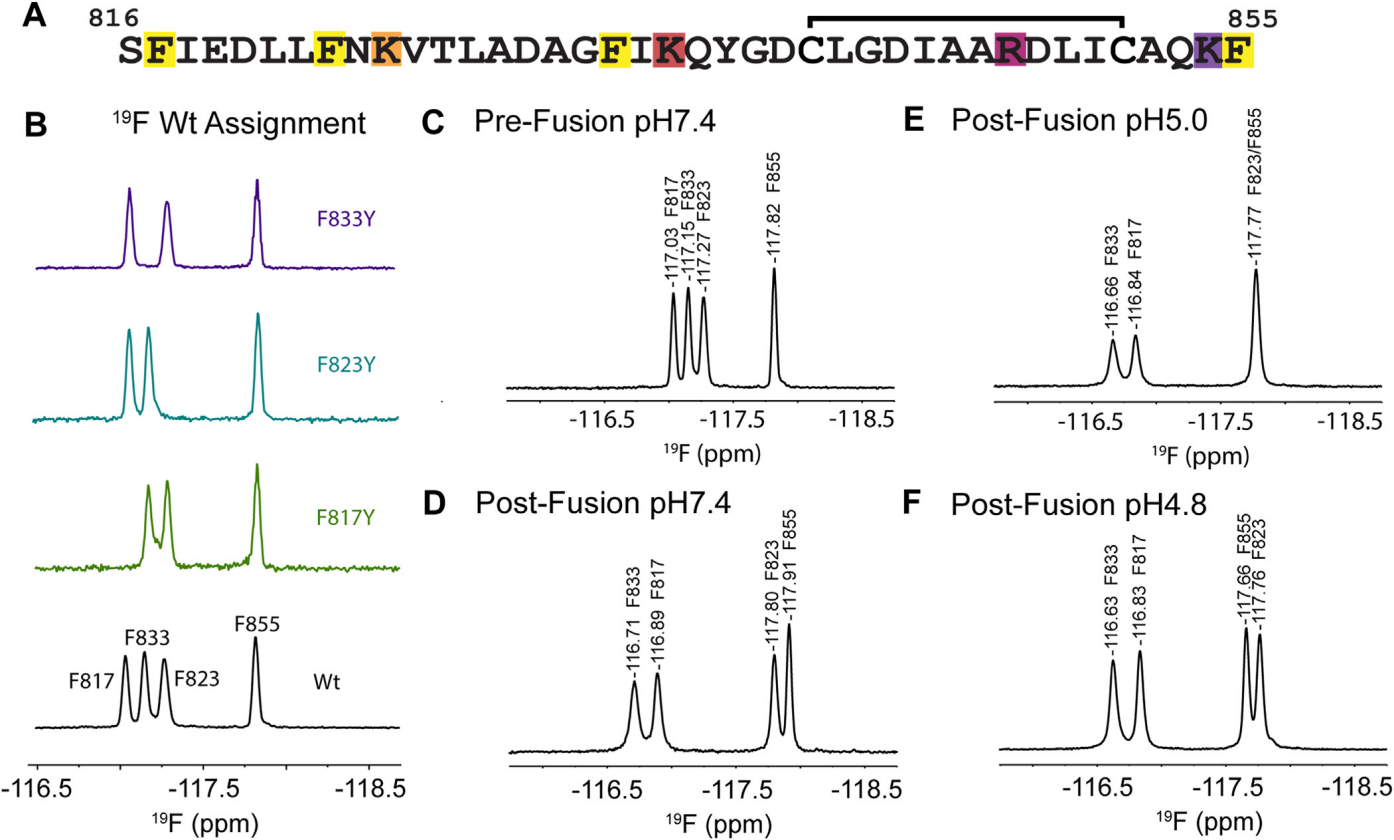
^**19**^**F-Phe Wt FD assignment and sensitivity to local environment.***A*, FD sequence with ^19^F labeled phenylalanine's (*Yellow*) and positively charged residues highlighted. *B*, a mutagenesis approach was utilized to assign the four ^19^F peaks, with each mutant resulting in the loss of a single peak corresponding to the missing phenylalanine. Clear chemical shifting is present as the FD goes from (*C*) a random coil in solution, (*D*) to a helix-turn-helix in the presence of DPC. The slight shifting from (*D*) pH 7.4 to (*E*) pH 5.0 correlates well with the minor conformational changes witnessed previously.^8^ (*E*) F823 and F855 overlap at pH 5.0, however, (*F*) a minor drop in pH can facilitate peak separation.

**Figure 4:**
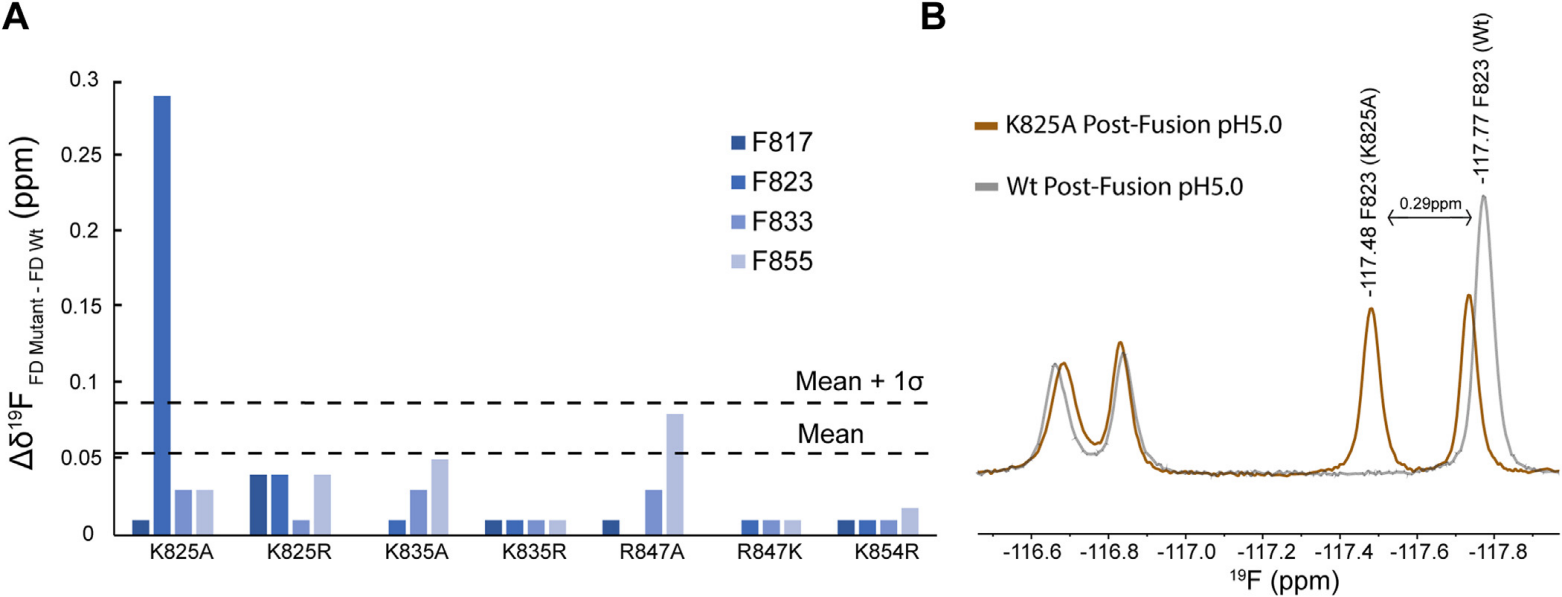
^**19**^**F-Phe structural analysis of FD mutants in DPC micelles.***A*, only K825A showed any significant CSPs, specifically for residue F823 found in helix one. *B*, overlaying the Wt and K825A ^19^F-Phe 1D spectra exhibits clear shifting of F823, with only marginal shifting for all other peaks.

To probe the structural nuances of the basic FD mutants further, a ^19^F NMR approach was utilized, through a ^19^F-Phe labeling scheme (20). The rationale is that through a deconvoluted ^19^F spectra, the local site of any structural perturbation could easily be identified. There are four phenylalanine residues within the FD (Fig. 3*A*), one near the N-terminus (F817), one at the C-terminus (F855), and two conveniently located in the helix-turn-helix motif found in the FP; F823 in helix one and F833 in helix two. The only other aromatic amino acid within the FD is a single tyrosine (Y837), which was successfully labeled alongside the four phenylalanine’s in the ^19^F-Phe+Tyr labeled Wt FD (Fig. S2). Unfortunately, no shifting for ^19^F-Y837 was witnessed between the functional states of the FD, likely due to the unique positioning of Y837 between the FP and FL. Each chemical shift was assigned to the correct corresponding residue *via* a mutagenesis approach, where three mutants were created (F817Y, F823Y, and F833Y), expressed with ^19^F-Phe, and analyzed (Fig. 3*B*). For example, with the F817Y mutant (Fig. 3*B* – Green) only three ^19^F-Phe peaks are observable such that the missing peak corresponds to F817 when compared to Wt. In the pre-fusion state, where the FD is a completely random coil (12), four individual and well-dispersed peaks are observed (Fig. 3*C*). Following the addition of DPC micelles, CSPs are witnessed for all peaks as the FD embeds within the micelle and forms the helix-turn-helix structure(Fig. 3*D*) (21). As expected F823 and F833 undergo the largest chemical shifts, with absolute CSPs of 0.53 ppm and 0.44 ppm respectively, as helix one and helix two are embedded in the post-fusion state. With CSPs of 0.15 ppm and 0.09 ppm, F817 and F855 undergo shifting to a much lesser extent indicating less significant structural changes in those regions. Minor shifts are then observable as we decrease the pH from pH 7.4 to pH 5.0 (Fig. 3, *D* and *E*), with F817 and F833 shifting 0.05 ppm, F823 0.03 ppm, and F855 0.14 ppm. This agrees well with the modest structural change discovered previously from the backbone assignment of the pH7.4 and pH5.0 Post-Fusion states (8). Due to the overlapping of residues F823 and F855 at 117.77 ppm in the Post-Fusion pH5.0 state, we decreased the pH to pH4.8 in order to separate the two. F855 is particularly sensitive to pH likely due to its C-terminal carboxylic acid group, displaying a CSP of 0.14 ppm from pH 7.4 to pH 5.0 and 0.10 ppm from pH 5.0 to pH 4.8, allowing us to discern the two peaks. The data shown in Figure 4 verifies the use of ^19^F-Phe labeling to detect structural changes within the FD at specific regions of interest such as the two helices in the helix-turn-helix motif.

Every basic amino acid mutant was labeled with ^19^F-Phe, expressed, and analyzed. All three states were captured (Pre-Fusion, Post-Fusion pH7.4, and Post-Fusion pH5.0) and compared to Wt to observe any structural perturbations. Our primary focus was the Post-Fusion pH5.0 state due to the increased functional relevance of the FD structure at low pH. CSPs compared to Wt were plotted (Fig. 4*A*) and displayed significant perturbations for F823 within the K825A mutant (Fig. 4*B*), suggesting the mutation impacts helix one. With little structural perturbations present we turned to investigate the sidechain dynamics, with spin-lattice (T1) and spin-spin (T2) experiments performed in the ^19^F dimension, however, little conclusions could be drawn. No significant deviation from Wt could be observed across any mutant, although differences in the dynamics of individual residues were detected (Fig. S3). Together, this data supports the use of ^19^F-Phe to identify the location of structural perturbations such as the local impact of K825A, from a simplified, deconvoluted spectra at atomic resolution.

**Figure 5:**
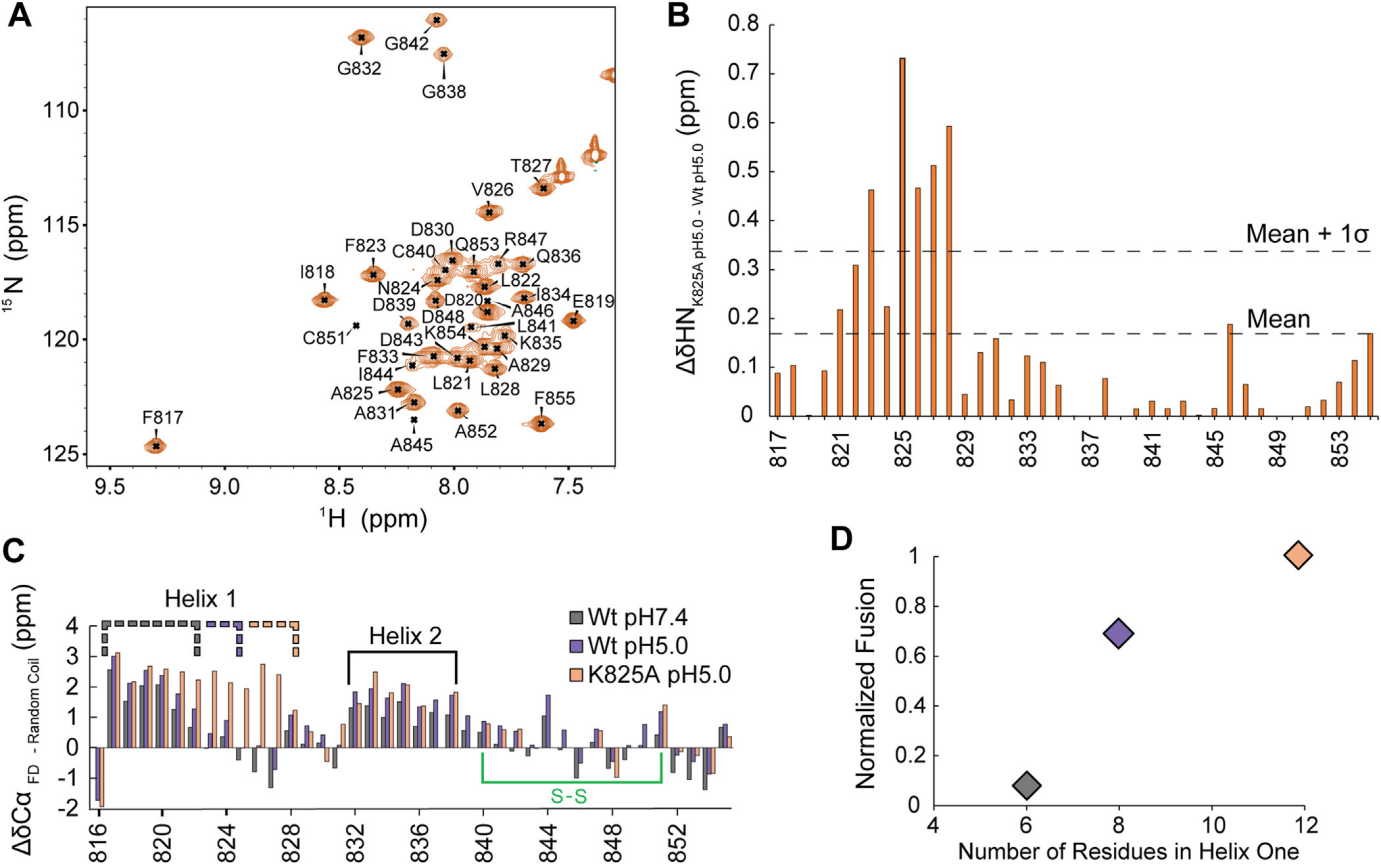
**K825A has an extended helix one which is heavily implicated in FD fusogenecity**. *A*, The backbone of K825A was assigned in DPC micelles (*B*) and CSPs were plotted, displaying perturbations within helix one only. *C*, K825A shows a helical elongation within helix one compared to the Wt Post-Fusion state at pH 7.4 and 5.0, (*D*) which correlates with an increase in fusion in 80:20 POPC:POPS liposomes. The Wt pH 7.4 (6 amino acids) fusion was acquired in 75:25 POPC:POPS liposomes due to low levels of fusion.

To confirm the conclusions gathered from the ^19^F data regarding K825A, a more traditional ^13^C ^15^N NMR labeling approach was utilized. All 40 residues within K825A were assigned and compared to Wt at pH5.0 (Fig. 5*A*) where several CSPs were found, particularly around the site of the mutation (Fig. 5*B*). This indicates that the mutation only significantly impacted helix one, in terms of the secondary structure, as predicted *via* the ^19^F sidechain data (Fig. 4). When plotting the chemical shift index (CSI) for K825A, a clear elongation of helix one is observable (Fig. 5*C*). Intriguingly, when comparing the length of helix one *via* CSI across Post-Fusion pH 7.4 (6 residues), Post-Fusion pH5.0 (8 residues), and K825A (12 residues) pH 5.0 to fusogenic activity, a positive correlation can be observed (Fig. 5*D*). In summary, helix one within the FP region of the FD has been elucidated as a key component in the initiation of membrane fusion. This is likely due to the embedding of the helix resulting in a significant perturbation of the local membrane environment, with this perturbation of lipids in the outer leaflet essential for lowering the energetic cost associated with the coalescing of opposing membranes.

## Discussion

During the process of viral entry, the SARS-CoV-2 spike protein displays multiple traits that stand out in comparison to other enveloped viruses, such as a secondary cleavage event (2, 22) and the ability to fuse within either the plasma or endosomal pathway (6, 23). Intriguingly, both of these traits are linked to the FD, a well-conserved stretch of 40 amino acids at the N-terminus of the cleaved S2 subunit. Previous work has implicated pH, and membrane lipid composition as key modulators of FD-mediated fusion, with the FD demonstrating a positive fusogenic relationship with low pH and increasing anionic lipid concentrations; two characteristics of the endosomal membrane (8, 9, 10, 23). Given these findings, the relationship between the FD and negatively charged lipids is one that deserves further attention in order to elucidate the molecular details by which the FD initiates viral fusion. Through a mutagenesis-based approach, the mechanism of SARS-CoV-2 FD initiated fusion has been further elucidated, with the importance of basic amino acids (K825, K835, R847, and K854) clarified, and helix one of the FP region-proven to be a key constituent within the fusion process.

Electrostatic interactions are not heavily associated with the role of viral FDs, predominately due to the necessity for such fusogenic regions to bury within the membrane, making charged residues rather counterintuitive due to the increased energy cost (24, 25). Astonishingly, the FD of SARS-CoV-2 contains 10 such charged residues (four positive and six negative), with high sequence conservation indicative of a critical role, which to date remains unclear. There is some evidence to suggest that each negative residue participates in the coordination of two Ca^2+^ ions (26). For clarity, Ca^2+^ was omitted from this study in order to focus on the interactions between the individual positive amino acids and membrane lipids, particularly as the role of the divalent cation is still unclear within the FDs fusion mechanism. Moreover, the possibility of a potential intra or inter-molecular salt bridge was also ruled out in a comparative study alongside the LASV FD without Ca^2+^ present (14). In a high salt environment, it was shown that the affinity and fusogenicity of the LASV FD with anionic membranes decreased significantly more than that of the SARS-CoV-2 FD due to the loss of an intra-molecular salt bridge. However, a decrease in the initial binding affinity and fusogenecity for the SARS-CoV-2 FD in a high salt environment was witnessed, suggesting that weaker electrostatic interactions may have been perturbed. The decreased binding affinity when the basic residues are mutated to alanine as opposed to conserving their positive charge (Fig. S1) and the lack of interaction witnessed in the absence of anionic lipid (10), suggests that transient electrostatic interactions could well be taking place between the SARS-CoV-2 FD and anionic membrane.

The importance of anionic lipids within FD-mediated fusion is apparent, thus implicating positively charged residues within the molecular mechanism (10). As the FD previously displayed a fusogenic preference for the endosomal resident anionic lipid BMP, it was pertinent to establish whether this was due to any specific interactions on an individual amino acid basis. Regardless of whether POPS or BMP was present, similar functional trends were witnessed amongst all mutants (Fig. 2*A*), suggesting that any protein-lipid interactions may not be specific to either anionic headgroup. The positive amino acids within the FD appear to be important for both the initial interaction between the FD and anionic liposomes (Fig. S1) as well as for fusion (Fig. 2*A*). Looking at the mutants individually, K835R (Helix two) and K854R (C-term) retain the most fusogenecity compared to Wt. The fact that K835R (Helix two) displays more fusion than K835A (Helix two) could indicate that some electrostatic interactions are maintained by the arginine mutant (Fig. 2*A*). With regard to K854 (C-term), as it exists so close to the c-terminus, the dynamic nature of the region could be augmenting the gathered data and thus, it is difficult to predict a mechanistic role for this residue. K825R (Helix one) and R847K (FL) displayed the greatest loss of fusion activity within the charge-conserving mutants, suggesting these residues perform highly specific functions within the fusion mechanism. One idea with regard to this function is the modulation of the local membrane environment in the form of membrane thinning. Simulations of the SARS-CoV-2 FD have provided evidence toward an ability to thin the local membrane, and even implicated charged residues within the mechanism of action (18). Perhaps the interactions between basic residues and the anionic membrane allow the FD to manipulate the lipid headgroups and induce negative curvature. This action labeled as polar head intrusion (27), has been proposed to catalyze fusion by reducing the repulsion of opposing membrane headgroups having been elucidated as an important component of the influenza fusion peptides activity (28, 29). This theory is further strengthened by the specific, positive relationship witnessed between the SARS-CoV-2 FD and the endosomal resident lipid BMP (10). The inverted conical shape of the lipid favors local negative curvature and promotes membrane fluidity, likely lowering the energetic barrier necessary for the FD to modulate the local membrane environment. Further research into the interactions of individual basic amino acids with negatively charged lipids and their resulting impact on the structure of the membrane is essential to prove this hypothesis.

In a simple model, we anticipate that the binding affinity of the FD to a membrane would positively correlate with fusion. This is intuitive as FD-mediated fusion cannot occur unless the FD comes into contact with the membrane in question. Curiously, the K825A (Helix 1) mutant displayed a significant decrease in binding affinity (Fig. S1) but an increase in fusion (Fig. 2). However, the molecular picture is not so straightforward. It is likely that more than one FD must interact within a localized area of the membrane to initiate fusion. This is due to a single FD being unable to elicit a significant enough perturbation on the membrane alone, and thus the gathering of several FDs on the membrane surface is required. With the α-helical elongation witnessed in K825A we believe that helix one would bury deeper within the membrane and thus the perturbing effect of an individual FD to be greater than that of the Wt. Hence, whilst Wt may interact more readily with the liposomes, K825A may elicit a significantly larger membrane perturbing effect, lowering the number of FDs needed to bind the membrane for a successful fusion event to occur. This hypothesis is reinforced by the increased fusion witnessed with K825A compared to Wt within the fusion assay when both FD and liposome concentration were fixed to 10 μM and 200 μM, respectively.

To try and understand the unique properties of K825A (Helix one), we investigated the structure of the mutant at atomic resolution in DPC micelles. Detergent micelles are often utilized for NMR studies due to their small size and relative ease of use, yet the dynamic nature and intrinsic instability of detergents can often be detrimental to protein structure and function. However, our previous studies have shown a similar helical propensity in vesicles as is witnessed in DPC micelles (12) and the Cα CSI of our Wt Post-Fusion pH5.0 state in a DPC micelle correlates well with that of the CSI of the structure solved in DMPC:DH7PC bicelles (PDB:7MY8) (21). While we are confident the secondary structure information acquired in DPC micelles closely mimics that witnessed in the more physiologically relevant bicelle, further studies utilizing nanodiscs with different lipid compositions could be beneficial to understand the structure of the FD within a more physiologically relevant membrane environment. The increased α-helicity of K825A was found to be a four amino acid elongation of helix one, through a combination of ^19^F-Phe CSPs and a more traditional ^13^C ^15^N backbone assignment. In the Wt at pH7.4, helix one terminates at L822, at pH5.0 the helix contains the full hydrophobic motif ‘^821^LLF^823^’ and terminates at N824, whilst in K825A the helix extends to L828. The highly conserved ‘LLF’ motif has been shown on multiple occasions to be critical for the fusogenecity of the FD, playing a key role in inserting the FD into a lipid environment alongside membrane ordering (13, 30, 31, 32). The modulation of ‘LLF’ within helix one appears to play a critical role in fusion, and its incorporation into helix one at pH5.0 is proposed to be a significant factor for the increased fusogenecity witnessed for the FD at low pH. It is less clear how ‘LLF’ may contribute to the increased fusion witnessed in K825A; however, CSPs are witnessed for the motif suggesting the chemical environment could be altered in a manner that is positive for fusogenecity. This could be in the form of a novel orientation, the formation of new interactions, or an increased depth of insertion. Moreover, the positive relationship found between increasing α-helicity and fusion witnessed (Fig. 5*D*) has been found on several occasions previously, with α-helices capable of embedding within and destabilizing different membrane environments (33, 34, 35, 36). Using a helical wheel projection, an amphipathic character can be witnessed in helix one when K825 is not incorporated such as in the Wt at pH5.0. However, replacing this residue with a non-polar residue such as alanine allows the helix to elongate whilst maintaining amphipathicity (Fig. S4). With polar residues energetically unfavorable within the hydrophobic core of the membrane, snorkeling is a common trait witnessed with charged residues. Snorkeling allows the aliphatic chain of the residue to exist within the membrane, whilst the charge group is placed amongst the polar lipid headgroups (37). Therefore in the Wt, K825 could be snorkeling to form electrostatic interactions in the presence of anionic lipid, which in turn may prevent the elongation of helix one. Similar interactions were predicted in K825R which maintains the same secondary structure as Wt (Fig. 2*B*), however a decreased binding affinity and fusogenecity compared suggests a more complicated explanation for the role of K825 than solely electrostatic interactions. This contrast may be explained by the increased strength by which the arginine sidechain interacts with lipids (38), potentially preventing the FP from inserting deeper or further restricting the movement of the FD within the membrane.

In conclusion, basic amino acids in the SARS-CoV-2 FD are important toward the mechanism of membrane perturbation that leads to the initiation of viral fusion. Even when the positive charges are conserved through mutagenesis, a significant loss of function remains present, suggesting a highly specialized role for those original residues. In particular, K825 and R847 have been elucidated as playing key roles within the fusion mechanism and are intriguingly found to be very well conserved throughout the β-coronavirus family. The significant functional implications of R847 alongside the natural occurrence of alanine in that position throughout the α, γ, and δ coronavirus families, suggests this residue could have contributed to the creation of the β-coronavirus lineage and/or a zoonotic jump at some point in time. Furthermore, if the gain of function mutant, K825A, occurred randomly in nature it could contribute towards a more fusogenic spike protein and a novel strain of SARS-CoV-2 with increased pathogenicity. As the most highly conserved of all basic amino acids within the coronavirus FD, it is surprising to discover a gain of function mutation at K825; however, currently, we cannot rule out an important role for the lysine residue within the pre-fusion conformation of the spike protein. It is also important to note that although a lysine-to-alanine mutation may be unlikely to randomly occur as two base pair mutations are necessary, other non-polar mutations at position 825 could elicit the same effect. Serine is already naturally found at the equivalent position within some avian members of the γ coronavirus family, and with only a single base pair mutation necessary to produce alanine, this mutation could result in a zoonotic jump. Future research must focus on further understanding the gain of function witnessed for K825A in the context of the full spike protein, alongside screening for other amino acids that may elicit the same effect. Assessing mutants within the FD from members of the γ coronavirus, such as the avian infectious bronchitis virus (IBV) would also be extremely pertinent.

## Experimental procedures

### Materials

All lipids were acquired from Avanti Polar Lipids in chloroform (POPC: 1-palmitoyl-2-oleoyl-glycero-3-phosphocholine, POPS: 1-palmitoyl-2-oleoyl-sn-glycero-3-phospho-L-serine, BMP: bis(monooleoylglycero)phosphate (S,R Isomer), LissRhod-PE: 1,2-dioleoyl-sn-glycero-3-phosphoethanolamine-N-(lissamine rhodamine B sulfonyl) and NBD-PE: 1,2-dioleoyl-sn-glycero-3-phosphoethanolamine-N-(7-nitro-2-1,3-benzoxadiazol-4-yl), except for the detergent Dodecyl phosphocholine (DPC) which was purchased from Anatrace. 4-Fluoro-DL-Phenylalanine (^19^F-Phe), 3-Fluoro-DL-Tyrosine (^19^F-Tyr), and glyphosate were acquired from Fisher Scientific, with traditional NMR isotopes (^13^C-glucose and ^15^N-ammonium chloride) purchased from Cambridge Isotopes.

### Expression of ^19^F-Phe Fluorinated fusion domain

The SARS-CoV-2 FD is made up of 40 amino acids (^816^SFIEDLLFNKVTLADAGFIKQYGDCLGDIAARDLICAQKF^855^), within which exists four phenylalanine, one tyrosine and zero tryptophan. The following ^19^F expression protocol was initially adapted from Mandala and co-workers (20), with unlabeled, and ^13^C ^15^N Expression described previously (12). A single colony from an LB plate was used to inoculate 25 ml of minimal media (pH 7.3, 56 mM Na_2_HPO_4_, 15 mM KH_2_PO_4_, 9 mM NaCl, 8 mM NH_4_Cl, 55 mM Glucose, 1 mM MgSO_4_, 0.3 mM Na_2_SO_4_ 0.2 mM CaCl_2_, trace amounts of thiamine and biotin, 50 mg kanamycin) and grown overnight (∼20 h) at 37 °C. The next morning the confluent starter culture was added to 1L of minimal media and grown at 37 °C until an OD_600_ of 0.5 was achieved (approx. 6 h). Subsequently, the temperature was lowered to 18 °C and 1.5 g of glyphosate was added to prevent aromatic amino acid biosynthesis through arresting the pentose phosphate pathway (39). This was followed by the addition of 115 mg L-Tryptophan, 115 mg L-Tyrosine (or 400 mg of 3-Fluoro-DL-Tyrosine), and 400 mg 4-Fluoro-DL-Phenylalanine. After 30 to 60 min, 1 mM IPTG was added, and protein expression proceeded at 18ᵒC overnight (∼20 h). Cells were then collected by centrifugation at 4000*g* for 30 min at 4 °C, with the resulting pellet either stored at −80 °C or purified immediately.

### Purification of the fusion domain

The purification of the SARS-CoV-2 FD has been described in detail previously and remains consistent regardless of the expression/labeling system (12). Briefly, 8M urea was used to solubilize the cell pellet followed by Ni-NTA affinity chromatography and cleavage *via* SUMO protease in dialysis. The cleaved FD is then isolated through the use of Ni-NTA affinity chromatography once again and dialyzed to remove reducing agents, ensuring the correct formation of the disulfide bond. Following dialysis, the sample was purified further *via* size exclusion chromatography (SEC) with 10 mM HMA 100 mM NaCl pH 7.4 as the mobile phase. Resulting fractions are pooled, concentration determined *via* A_280_, and stored at 4 °C.

### Preparation of small and large unilamellar vesicles

Specific amounts of lipids solubilized in chloroform were added to glass test tubes *via* a Hamilton syringe. Chloroform was then removed *via* gentle vortexing whilst applying a continuous stream of nitrogen to create a lipid film, followed by storage in a desiccator under vacuum overnight. For small unilamellar vesicles (SUVs), the lipid film was resuspended in the chosen buffer *via* vortex, and sonicated for 15 min at 10% duty cycle (1s on/1s off) using a Branson ultrasonicator microtip with the sample sat in ice water. The SUVs were then centrifuged at 20,000*g* for 10 min to remove residual metal particulates. For large unilamellar vesicles (LUVs), the lipid film was resuspended in buffer and vortexed extensively. The lipid suspension was subjected to 10 freeze-thaw cycles between liquid nitrogen and a 42 °C water bath. Liposomes were extruded using a liposofast extrusion kit (Avestin) a total of 21 times through 2 polycarbonate membranes with a 100 nm pore size. All vesicles were either used immediately or stored for a maximum of 72 h at 4 °C prior to use.

### NMR experiments

NMR spectra were acquired using a Shigemi NMR tube in 10 mM Hepes/MES/Sodium Acetate (HMA), 100 mM NaCl, pH 7.4 or pH 5.0 buffer with 90%H2O/10%D2O. DPC was present in all Post-Fusion states as a membrane mimic at a concentration of ∼100 mM. All experiments were carried out on the Bruker Ultrashield 600 MHz magnet with a CP2.1 TCI 600S3 H&F/C/N-D-05 Z XT at a temperature of 23 °C. Typical ^19^F experiments consisted of 2000 scans, an acquisition time of 0.15s, D1 of 0.3s, and a sweep width of 20 ppm. All ^19^F data was processed using the software Mestrenova (40), and corrected to an internal TFA standard at −76.55 ppm. For the backbone assignment of K825A, HNCA, HN(CO)CA, HNCO and HN(CA)CO experiments were performed, and processing was carried out through SPARKY((41)) *via* NMR Box (42).

### Circular Dichroism (CD) spectroscopy

CD data was acquired *via* a Jasco J810 Spectro-Polarimeter using a quartz cuvette with a 2 mm path length. All experiments were carried out at room temperature (∼23 °C) in 1 mM HMA, 10 mM NaCl, 50 mM DPC, pH 5.0 with a protein concentration of ∼20 μM. Data was collected from 260 nm to 190 nm with a step size of 1 nm at 50 nm/min and averaged over three accumulations. Baselines were acquired in the exact same buffer, without any protein present, and subtracted from all data.

### Lipid mixing assay

Unlabeled LUVs composed of specified lipids were mixed with labeled LUVs containing the same composition with the addition of 1 mol% of the fluorescent labels: LissRhod-PE and NBD-PE. Experiments were carried out at a ratio of 9:1 unlabeled: labeled liposomes in Corning Costar black-walled, clear bottom 96-well plates with excitation and emission wavelengths at 460 nm and 538 nm respectively, with a 530 nm cut-off. A SpectraMax M5 microplate at 23 °C was used to record the fluorescence measurements. Percent fusion was calculated as $\frac{\left(I_{F}-I_{B}\right)}{\left(I_{100}-I_{B}\right)}$, where I_B_ is the initial background fluorescence, I_F_ is fluorescence intensity measured after decreasing the pH, and I_100_ is the 100% fluorescence intensity value gathered after complete vesicle rupture following the addition of 1% Triton X-100. Experiments were performed at pH5.0 with a peptide/lipid ratio of 0.05 (5 μM and 100 μM). Negative controls identical to the experimental wells except no protein was present, were run alongside all conditions and subtracted from the final values.

### Isothermal titration calorimetry (ITC)

To ensure sufficient buffer matching the FD sample was dialyzed against 4L of 10 mM Sodium Acetate, 100 mM NaCl at pH5.0 for 2 h at 4 °C. The same dialysis buffer was used to resuspend the lipid film prior to forming SUVs, which were composed of POPC:POPS 50:50 mol%. All samples were centrifuged at 20,000*g* for 10 min and degassed prior to each ITC experiment. All measurements were taken using a Malvern VP-ITC microcalorimeter with the following experimental parameters in place: initial delay: 1200s, 41 injections (1 × 2 uL and 40 × 6 uL), spacing: 300s, duration: 14.4s, Stir Speed: 270 rpm, ref power: 2 μcal/sec and temperature: 22 ^°^C. All processing was conducted through NITPIC (43), and SEDPHAT (44), with thermograms generated through GUSSI (45).

## Data availability

All data is located within this manuscript or the accompanying supporting information, with 10.13039/501100004182NMR data also submitted to the BMRB. SARS-CoV-2 Wt FD BMRB ID: 52445, SARS-CoV-2 K825A FD: 52446.

## Supporting information

This article contains supporting information.

## Conflict of interest

The authors declare that they have no conflicts of interest with the contents of this article.
