# Supplementary material for: Positive residues of the SARS-CoV-2 fusion domain are key contributors to the initiation of membrane fusion
Source: J Biol Chem. 2024 Jul 11;300(8):107564. doi: 10.1016/j.jbc.2024.107564 (PMC11357847; doi:10.1016/j.jbc.2024.107564)
Supplement: Supporting Figures [file mmc1.docx]

Supporting Information

Positive Residues of the SARS-CoV-2 Fusion Domain are Key Contributors to the Initiation of Membrane Fusion

Daniel Birtles, Lijon Guiyab, Wafa Abbas, Jinwoo Lee*

Department of Chemistry and Biochemistry, University of Maryland, College Park 20742, Maryland, USA

*Email: jinwoo@umd.edu

Figure S1: FD mutants interacting with 50:50 POPC:POPS liposomes in comparison to Wt. Example isotherms are displayed for (A) R847A and (B) K835R. (C) Perturbations in affinity are observable for all alanine mutants. (D) Whereas the charge conserving mutants all display binding of the same magnitude.


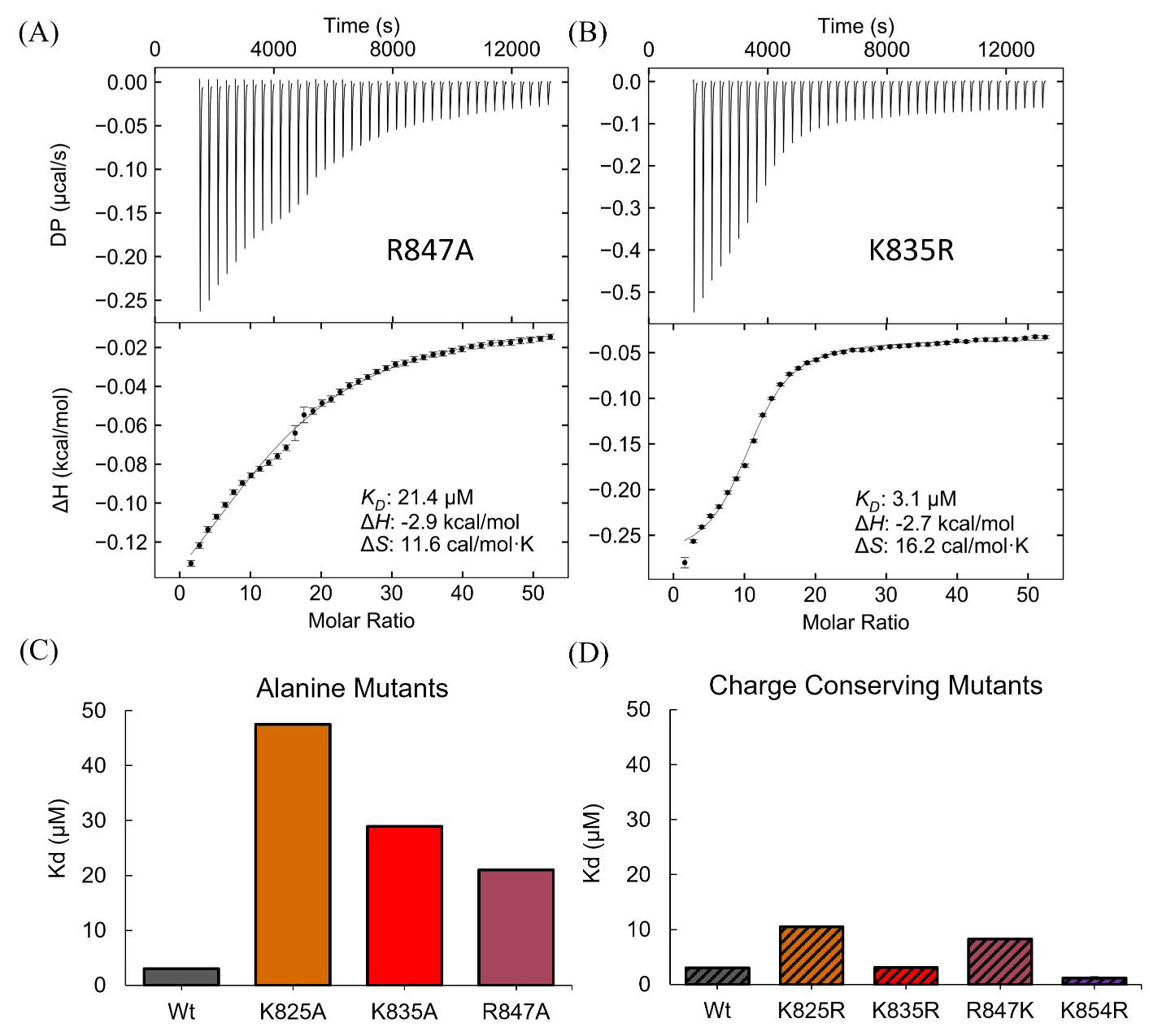

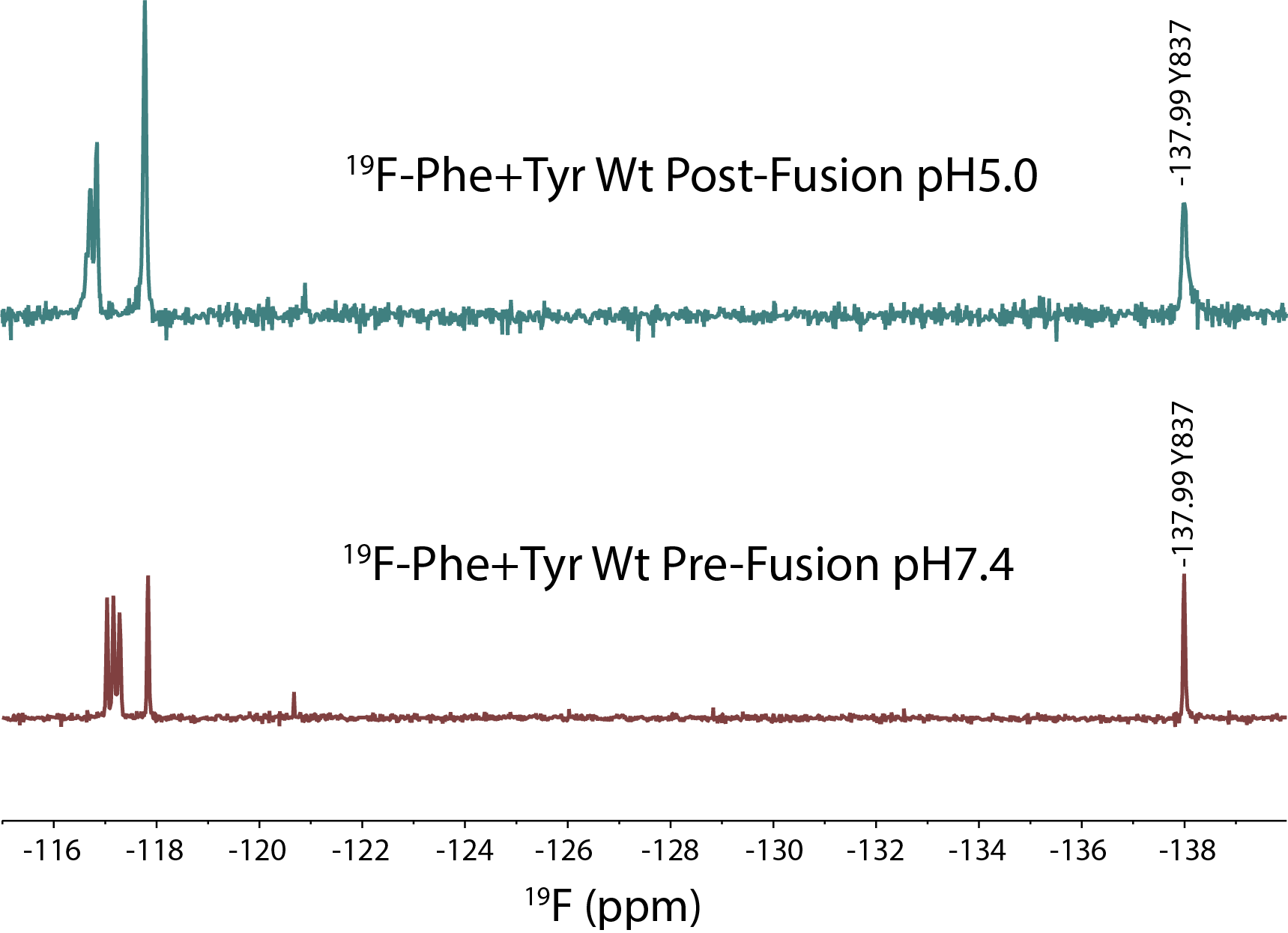


Figure S2: Phenylalanine and Tyrosine residues can be ^19^F labelled simultaneously. However, ^19^F-Y837 displayed no change in chemical shift and thus could not provide any further structural information. Post-Fusion refers to the FD being in a DPC micelle environment.

Figure S3: ^19^F dynamics. Spin-spin (T1) and Spin-lattice (T2) measurements were taken for the Wt and all mutants in DPC micelles, however little deviation was observed. Wt pH4.8 was analyzed due to the splitting of F823 and F855 highlighted in Figure 3, allowing the analysis of individual residues displaying no substantial difference from Wt pH5.0.


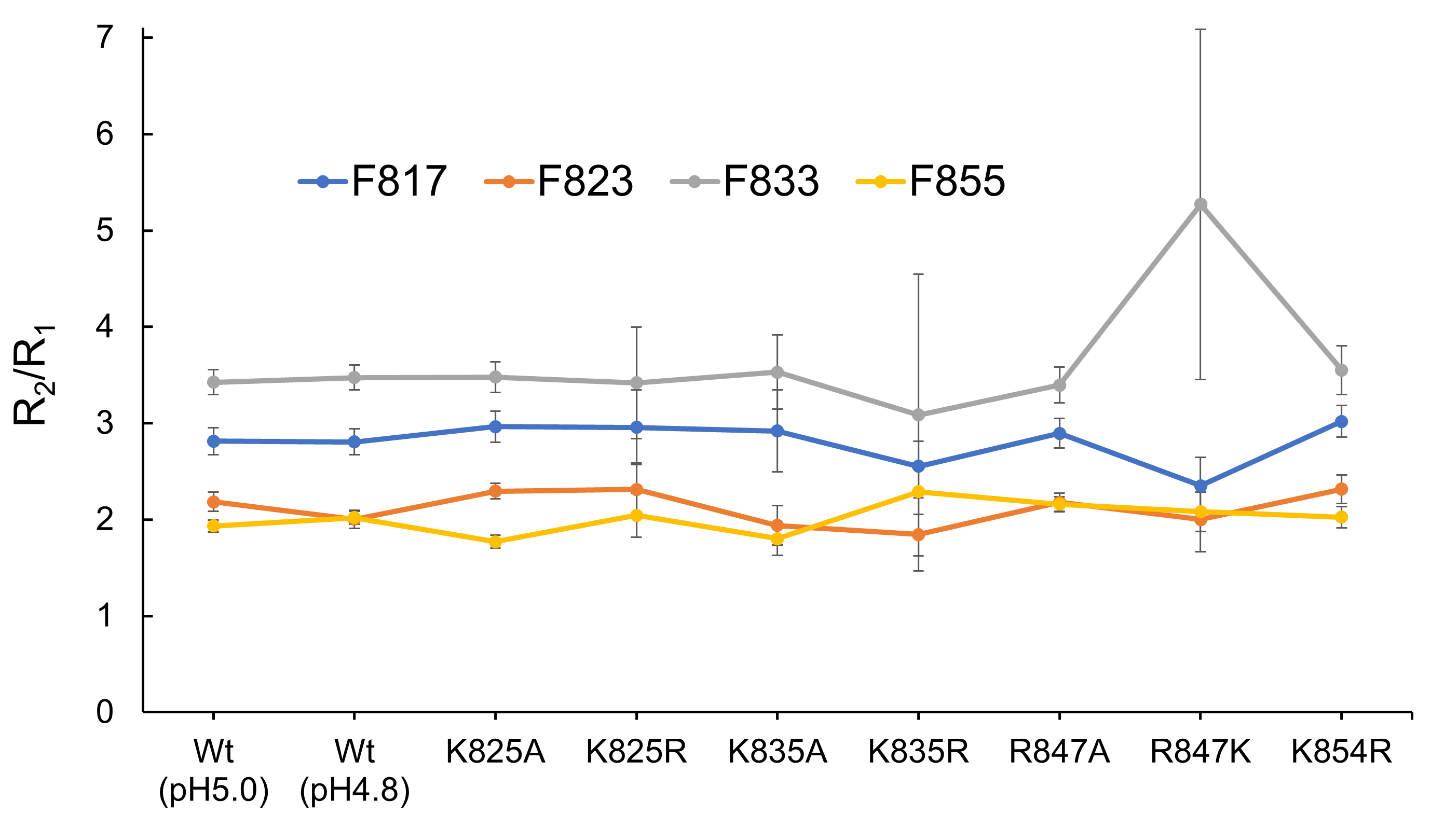

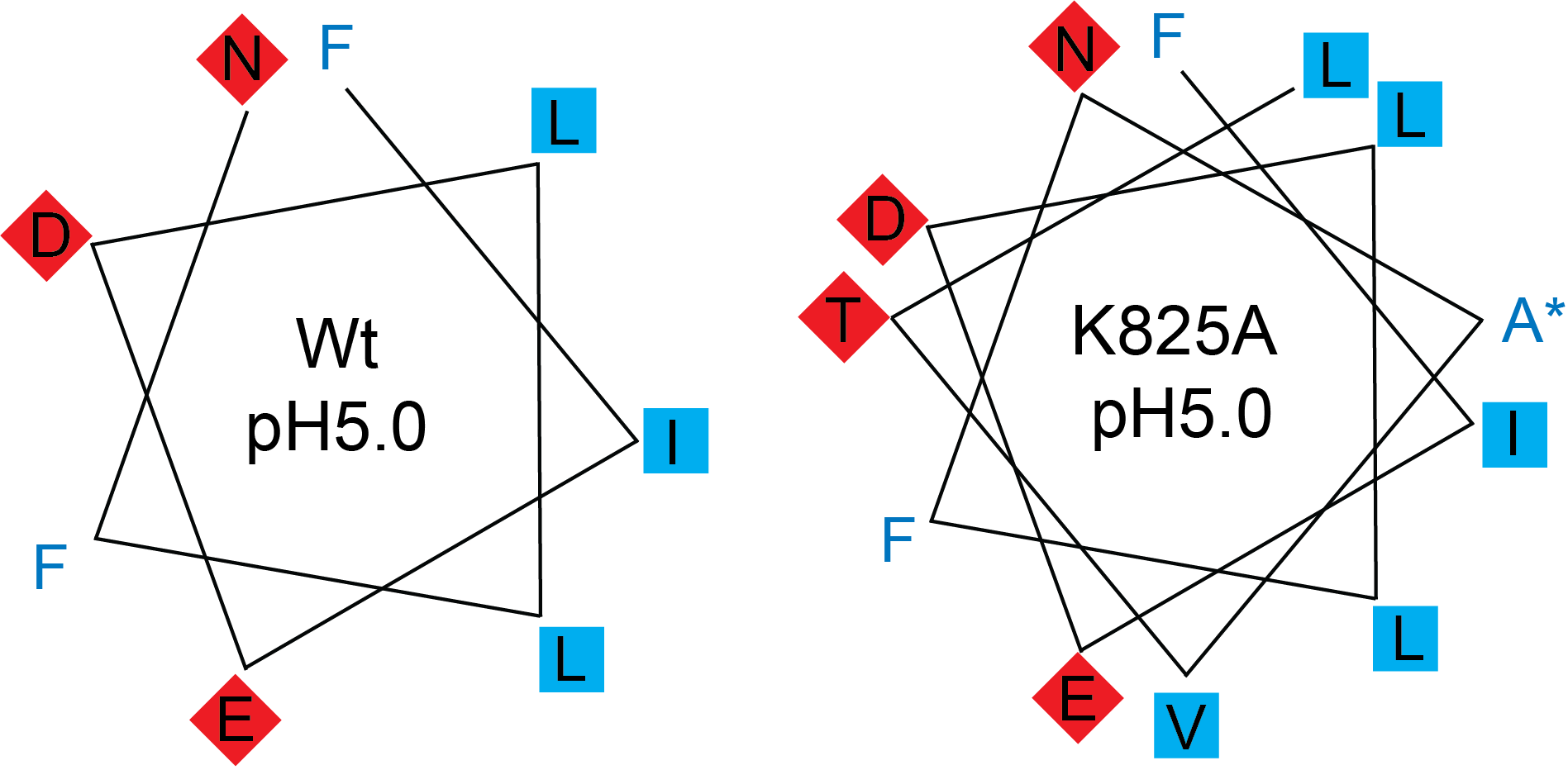


Figure S4: Helical wheel projection for helix one in Wt and K825A at pH5.0 in a DPC micelle environment. Amphipathicity is maintained following the helical elongation in K825A only due the mutation present. The site of K825A is denoted by *.
